# Bilateral Foot-Drop Secondary to Axonal Neuropathy in a Tuberculosis Patient With Co-Infection of COVID-19: A Case Report

**DOI:** 10.7759/cureus.11734

**Published:** 2020-11-28

**Authors:** Mohammad H AlKhateeb, Afia Aziz, Mugahid Eltahir, Abdulnasser Elzouki

**Affiliations:** 1 Internal Medicine, Hamad Medical Corporation, Doha, QAT

**Keywords:** covid-19, novel coronavirus, sars-cov-2 infection, tuberculosis, foot drop, peripheral neuropathy

## Abstract

Tuberculosis (TB) is a global pandemic and is one of the top 10 causes of death worldwide as well as the leading cause of death from a single infectious agent.^ ^It can cause a wide array of complications including peripheral neuropathy. In addition to TB pandemic the recent pandemic of coronavirus disease 2019 (COVID-19) has led to an increased interest in the co-infection of TB patients and COVID-19 and whether TB increases risk for COVID-19 and its role in causing severity of disease and vice-versa.^ ^This case report discusses about a young cachectic man who was found to have bilateral foot-drop under the setting of TB with co-infection of COVID-19 later confirmed to be axonal neuropathy on nerve conduction study. The report highlights the importance of differential diagnosis of TB in COVID-19 patients as well as the consideration of TB in a patient with peripheral neuropathy after nutritional causes have been ruled out.

## Introduction

Tuberculosis (TB) has been a slow pandemic causing major infection-related morbidity and mortality [1]. It has long been known to cause various extra-pulmonary manifestations and can affect any part of the body, one such rarer manifestation that has been associated with it is peripheral neuropathy. How it affects the peripheral nerves has not been studied in great detail; various mechanisms have been linked to this including direct invasion, immune-mediated phenomenon, vasa vasorum inflammation, and effect of anti-TB medications, but due to the rarity of this condition limited work has been done on this topic [2-3].

In addition, with the recent pandemic of COVID-19, several studies have come forth regarding the co-infection of TB with COVID-19 with the first set of studies suggesting the synergistic potential of COVID-19 with TB worsening prognosis of TB patients [4-5].

We present a case of a 28-year-old male diagnosed with miliary TB after developing peripheral neuropathy in the setting of COVID-19.

## Case presentation

A 28-year-old South Asian male with no relevant past medical history presented with six months history of dry cough and fever. His cough was worse at night, associated with night sweats and 16 kg unintentional weight loss. It had worsened dramatically with shortness of breath on mild exertion and fever with chills 15 days prior to the patient seeking medical attention. He also noticed difficulty in taking steps around one month ago that progressively worsened to involve weakness in lower limbs around the same time his cough worsened.

**Figure 1:**
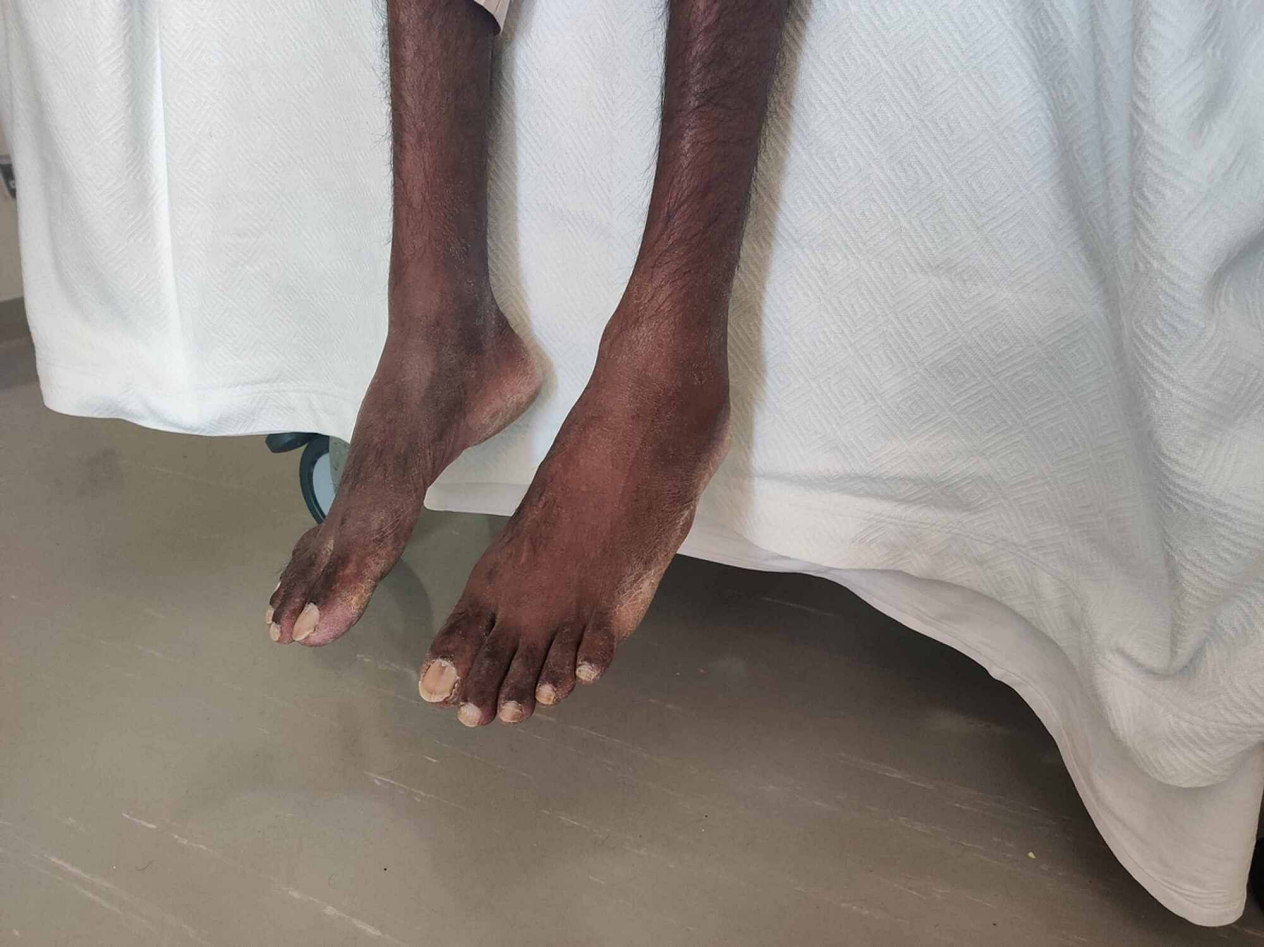
Bilateral foot-drop.

On examination, he was found to have generalized muscle wasting and was cachectic, he had bilateral foot-drop (Figure 1) as he was walking with a high steppage gait. He also had clubbing with left axillary and supraclavicular lymphadenopathy. The chest exam revealed reduced air entry on the left side with crepitations in the right lung base. Neurological exam initially was significant for loss of dorsiflexion and weak plantar flexion bilaterally with preserved ankle reflexes. Throughout his hospital stay, his neuropathy progressed with further loss of plantar flexion and ankle reflex with additional loss of pain sensation in his feet.

**Figure 2:**
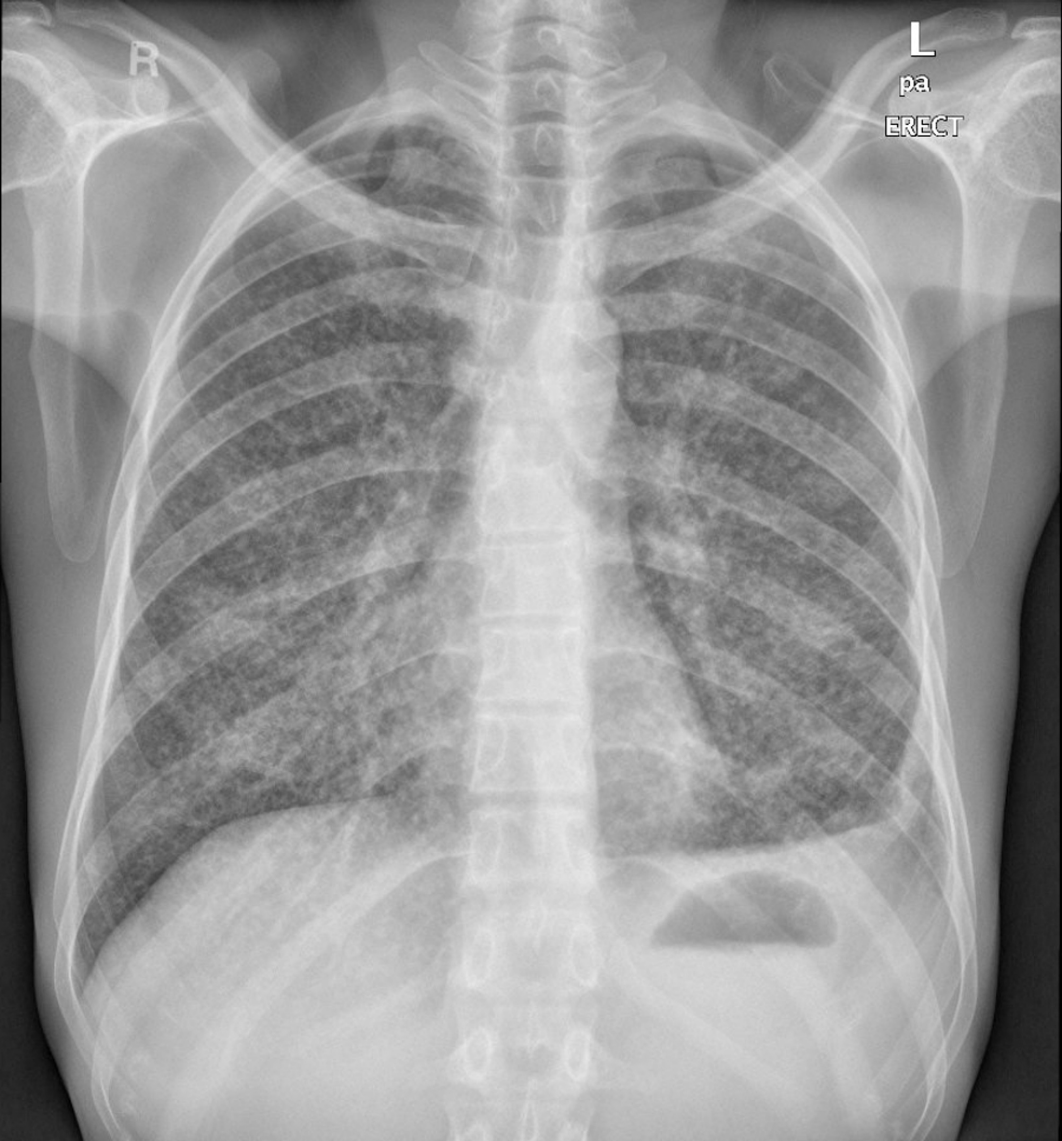
Chest X-ray showing bilateral multiple small nodules scattered in the lung fields giving the military shadow with blunting left costophrenic angle.

**Figure 3:**
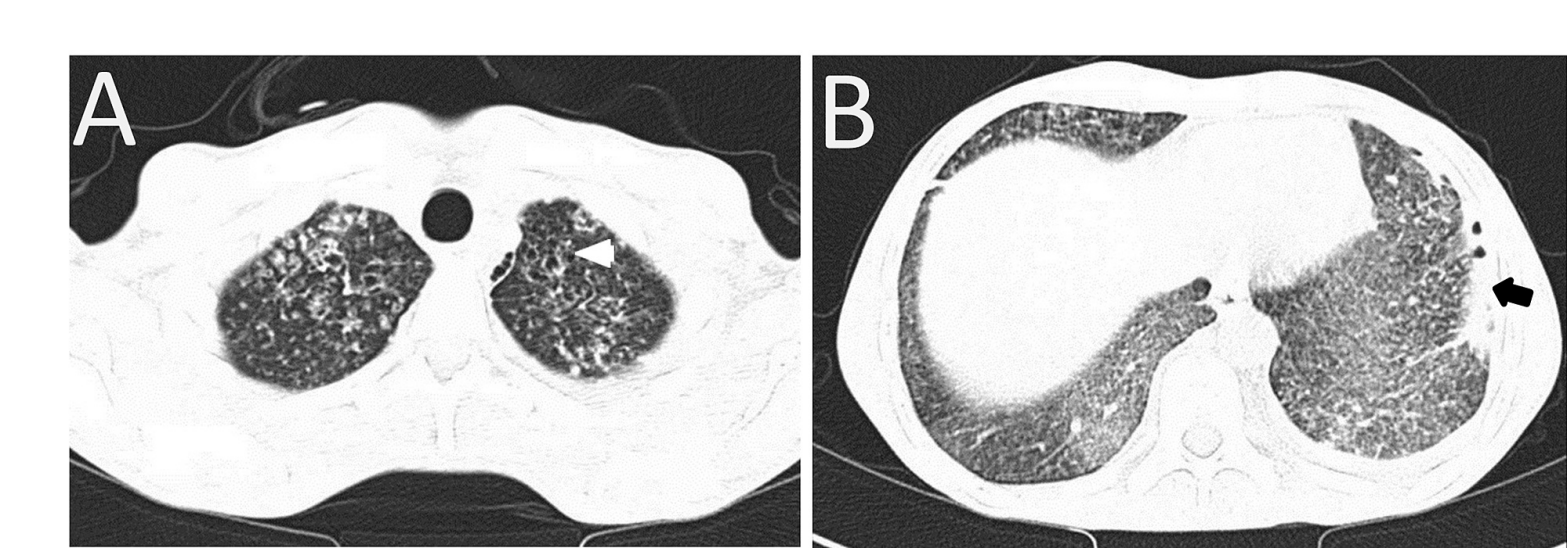
High resolution CT-scan showing diffuse innumerable miliary nodules. A. Cystic bronchiectatic changes in upper lobes (arrowhead). B. Left side mild pleural effusion with multiple air fluid level (arrow).

The patient was admitted to an isolation unit; first set of COVID testing was positive detected by reverse transcription-polymerase chain reaction (RT-PCR), his chest X-ray showed miliary shadowing (Figure 2). Additionally, he had hypochromic microcytic anemia and lymphocytopenia (Table 1). The patient was initiated on COVID protocol. Further evaluation showed vitamin B12 deficiency with iron stores depletion, elevated serum ferritin, and D-dimer level. HIV was nonreactive. Assessment for TB started with QuantiFERON and sputum for TB (i.e., acid fast bacilli and TB PCR), the patient was treated empirically initially with anti-TB therapy due to high clinical suspicion and chest X-ray findings despite the first set of sputum testing negative for acid fast bacilli. Mycobacterium tuberculosis was detected by PCR after repeated sets of sputum samples as bronchoscopy was delayed due to COVID-19 pandemic concerns. High resolution CT of the chest showed typical features for miliary TB (Figure 3).

**Table 1:** Laboratory findings during patient’s hospital stay. WBC, white blood cell; CRP, C-reactive protein; AST, aspartate transaminase; ALT, alanine transaminase; ALP, alkaline phosphatase; LDH, lactate dehydrogenase; INR, international normalized ratio; TIBC, total iron-binding capacity

Laboratory tests	Day 1	Day 2	Day 15	Normal range
WBC count	8.5	8.2	11.4	4–10 × 10^9^/L
Lymphocyte	0.9	0.4	1	1–3 × 10^9^/L
Hemoglobin	10.1	9.9	9.6	13-17 gm/dL
Platelets	398	351	425	150–400 × 10^3^/µL
CRP	27	34.5	50	0–5 mg/L
Lactic acid	2.5	–	–	0.5–2.2 mmol/L
Urea	3.8	2.8	4.5	2.8–8.1 mmol/L
Creatinine	50	39	34	62–106 umol/L
Sodium	128	135	131	136–145 mmol/L
Potassium	4.5	3.5	3.4	3.5-5.1 mmol/L
AST	44	46	80	0–41 U/L
ALT	61	37	84	0–40 U/L
ALP	49	52	134	13–53 U/L
Bilirubin	15	9	15	0–21 umol/L
LDH	443	–	–	135–225 U/L
INR	1.2	–	–	1
D-dimer	–	2.62	–	0–0.49 mg/L
Ferritin	1668	1651	3624	30–490 ug/L
Iron	–	2	–	6–35 umol/L
TIBC	–	23	–	45–80 umol/L
Transferrin	–	0.9	–	2–3.6 g/L
Creatine kinase	–	78	–	39–308 U/L
Vitamin B12	–	73.8	>1476	145–596 pmol/L

Peripheral neuropathy initially was attributed to vitamin B12 deficiency, but vitamin B12 replacement showed no improvement instead there was progression of his neuropathy. MRI spine did not show intraspinal, spinal cord, or thecal abnormalities. Nerve conduction study/electromyography (NCS/EMG) done at the time showed severe active sensorimotor axonal distal polyneuropathy with the involvement of bilateral posterior tibial and peroneal motor nerves, severe involvement of bilateral superficial peroneal sensory nerves, and mild involvement of right median and ulnar palmer sensory nerves and bilateral sural sensory nerves (Table 2). 

**Table 2:** EMG findings. Needle exam of selected muscles showed spontaneous activity with neurogenic changes more in lower than upper extremities and more distally than proximally. EMG, electromyography; INS ACT, insertional activity; FIB, fibrillations; PSW, positive sharp wave; FASC, fasciculations; MUP, motor unit potential; POLY, polyphasic units; AMP, amplitude; DUR, duration; RECR, recruitment; Int Patt, interferens pattern; N, normal.

Muscle	Side	INS ACT	FIB	PSW	FAS	MYO Disc	Normal MUP	POLY	Low AMP	High AMP	DUR	RECR	Int Patt
Deltoid	Right	Normal	0	0	0	0	+3	N	0	+1	Normal	Full	Full
First dorsal interosseous	Right	Normal	0	+1	0	0	+1	N	0	+1	Long	Full	Full
Vastus lateralis	Right	Normal	+1	+2	0	0	+1	+1	0	+1	Long	Reduce	Reduce
Tibialis anterior	Left	Normal	+2	+4	0	0	+1	+1	0	0	Normal	Reduce	Reduce
Tibialis anterior	Right	Normal	+2	+4	0	0	+1	+1	0	+2	Long	Reduce	Reduce
Gastrocnemius. Medial head	Right	Normal	+2	+3	0	0	+1	++	0	+2	Long	Reduce	Reduce
Gastrocnemius. Lateral head	Right	Normal	+2	+3	0	0	+1	++	0	+2	Long	Reduce	Reduce
Gastrocnemius. Medial head	Left	Normal	+2	+3	0	0	+1	+1	0	0	Long	Reduce	Reduce

The patient was discharged after getting one month of anti-TB treatment and kept on close follow-up. At six weeks he was contacted over the phone (using telehealth consultation) and as per him his gait and sensations had improved and he could now move his foot upwards. Unfortunately, he was lost to follow-up at eight weeks as he had left the country for good.

## Discussion

Since COVID-19 being declared as a global pandemic [6], co-infection with another pandemic as TB [1] was inevitable. Co-infection case reports have been on the rise [7-11], reaching to a cohort of 69 cases in the literature [5], describing respiratory symptoms almost exclusively without neurological involvement aside from one case that reported TB meningitis [12]. Our patient is an interesting case to report as he had a very rare form of neurological manifestation associated with his miliary TB that is axonal sensorimotor polyneuropathy causing bilateral foot-drop in the background of COVID-19 infection (whether COVID-19 was a confounding factor or synergistic one is unclear due to limited knowledge of this new disease) and malnutrition.

Polyneuropathy is a diffuse peripheral nerve disorder that is not confined to the distribution of a single nerve or a single limb and is relatively symmetrical bilaterally and can be secondary to myelin dysfunction, vasa nervosum compromise, or axonopathy. Causes of axonal polyneuropathy are broad and include a wide variety of conditions including but not limited to diabetes mellitus; uremia; alcohol; nutritional deficiencies including vitamin B12, copper, thiamine, folate, etc; toxins including chemotherapeutic agents, linezolid, amiodarone, isoniazid, etc.; infectious causes including leprosy, lyme disease, and HIV [13].

Even though neurological manifestations of TB have been well documented throughout history in the form of central nervous system involvement such as tuberculous meningitis, tuberculomas and brain abscess, rare manifestations of TB that have been reported are peripheral nervous system involvement in the form of compression by vertebral collapse (Pott’s disease) [14], and nerve compression by granulomatous tissue [15]. Still rarer forms include axonal sensorimotor polyneuropathy with only a handful of cases reported in literature attributing to variable mechanism: vasculitis, granulomas, and delayed hypersensitivity response [16-18]. Unfortunately, no such details could be isolated from this patient as a peripheral nerve biopsy was not done at the time due to COVID related concerns.

Nutritional causes were taken into consideration with his systemic disease and malnourished picture. But the patient lacked the typical features for vitamin B12 and thiamine deficiency as he had sparing of proprioception without ataxia or ophthalmoplegia [13]. Moreover, vitamin B12 deficiency usually presents as large fiber, sensory myeloneuropathy [19], unlike the nerve conduction study findings of the patient. In addition to his atypical clinical picture, he showed no improvement on prolonged course of vitamin B12 which was initially given in parenteral form then shifted to oral.

Isolated peripheral neuropathy is regularly described in TB patients after receiving anti-TB therapy, which is often responsive to pyridoxine and by discontinuing the offending agent (usually isoniazid) [13]; unlike our case who suffered from neuropathy prior to initiation of anti-TB therapy. 

HIV and hepatitis infections have been linked with axonal neuropathy [6] but they were tested negative in our patient. COVID-19 involvement of peripheral nervous system has been mostly linked with Guillain-Barre syndrome in which initial data report symptoms in the next two weeks following onset of COVID infection, with acute motor and sensory axonal neuropathy variant seen in almost one-fourth of cases [20]. Delayed response to anti-TB therapy (greater than eight weeks) in our case might suggest confounding etiology. It is still unclear whether COVID-19 had a direct impact or just a correlation.

## Conclusions

Peripheral neuropathy could have a wide variety of causes and TB, the great mimicker of diseases should also be taken into consideration after exhausting the other common potential causes of neuropathy and for avoiding misdiagnosis. Moreover, there is still a considerable need for studies regarding the potential impact of COVID-19 on TB, regarding prognosis and severity of disease as well as the underlying mechanism by which TB affects extra-pulmonary structures, especially the peripheral nerves.
